# Inferring the Phylogenetic Positions of Two Fig Wasp Subfamilies of Epichrysomallinae and Sycophaginae Using Transcriptomes and Mitochondrial Data

**DOI:** 10.3390/life11010040

**Published:** 2021-01-11

**Authors:** Dan Zhao, Zhaozhe Xin, Hongxia Hou, Yi Zhou, Jianxia Wang, Jinhua Xiao, Dawei Huang

**Affiliations:** Institute of Entomology, College of Life Sciences, Nankai University, Tianjin 300071, China; 2120181038@mail.nankai.edu.cn (D.Z.); 1120180392@mail.nankai.edu.cn (Z.X.); 1120180393@mail.nankai.edu.cn (H.H.); 1120170366@mail.nankai.edu.cn (Y.Z.); 1120170365@mail.nankai.edu.cn (J.W.)

**Keywords:** fig wasps, classification, phylogeny, mitochondrial gene, transcriptome

## Abstract

Fig wasps are a group of insects (Hymenoptera: Chalcidoidea) that live in the compact syconia of fig trees (Moraceae: *Ficus*). Accurate classification and phylogenetic results are very important for studies of fig wasps, but the taxonomic statuses of some fig wasps, especially the non-pollinating subfamilies are difficult to determine, such as Epichrysomallinae and Sycophaginae. To resolve the taxonomic statuses of Epichrysomallinae and Sycophaginae, we obtained transcriptomes and mitochondrial genome (mitogenome) data for four species of fig wasps. These newly added data were combined with the data of 13 wasps (data on 11 fig wasp species were from our laboratory and two wasp species were download from NCBI). Based on the transcriptome and genome data, we obtained 145 single-copy orthologous (SCO) genes in 17 wasp species, and based on mitogenome data, we obtained 13 mitochondrial protein-coding genes (PCGs) for each of the 17 wasp species. Ultimately, we used 145 SCO genes, 13 mitochondrial PCGs and combined SCO genes and mitochondrial genes data to reconstruct the phylogenies of fig wasps using both maximum likelihood (ML) and Bayesian inference (BI) analyses. Our results suggest that both Epichrysomallinae and Sycophaginae are more closely related to Agaonidae with a high statistical support.

## 1. Introduction

Fig wasps (Hymenoptera: Chalcidoidea) refer to all wasps that must rely on the syconia of fig trees (Moraceae: *Ficus*) to complete their life histories. According to whether they pollinate the figs, fig wasps are broadly classified into two categories of pollinating fig wasps and non-pollinating fig wasps [1]. Among them, the symbiosis of figs–pollinating fig wasps is a classical model of the mutualistic system, which originated about 75 million years ago [2]. The plant–insect interaction system between figs and fig wasps provides an ideal model for the study of the co-evolution of species and symbiotic relationship among organisms [3], and these studies are inseparable from the correct identification and phylogenetic history reconstruction of fig wasps.

The current taxonomic information listed on the fig web (http://www.figweb.org/Fig_wasps/Classification/index.htm) is mainly based on the taxonomy system proposed by Heraty [4], in which the fig wasps are classified into five families (Agaonidae, Eurytomidae, Ormyridae, Pteromalidae, Torymidae) and ten subfamilies (Agaoninae, Kradibiinae, Sycophaginae, Tetrapusiinae, Colotrechinae, Pteromalinae, Epichrysomallinae, Otitesellinae, Sycoecinae, and Sycoryctinae) [4]. The taxonomic statuses of some subfamilies are difficult to determine, such as the non-pollinating wasps of Epichrysomallinae and Sycophaginae [5,6,7,8].

The taxonomic history of Epichrysomallinae and Sycophaginae has undergone multiple changes since the beginning of the taxonomy of fig wasps in the 19th century. Sycophaginae was founded by Walker in 1875 [9], then it was included in the family of Torymidae based on cleptoparasitic habits by Joseph in 1964 [10]. Epichrysomallinae was established and classified in Torymidae by Hill in 1967, who also agreed to classify Sycophaginae in Torymidae [5]. In 1981, Bouček revised and adjusted Epichrysomallinae into Pteromalidae on the basis of morphological characteristics [6]. In 1988, Bouček further considered the whole Agaonidae as a monophyletic group based on the reproductive characteristics, of which the Agaoninae was only the most specific group with the behaviors of pollination, and thus both Epichrysomallinae and Sycophaginae were classified in Agaonidae [7]. In 1998, Rasplus et al. used the D1 and D2 domains of nuclear 28S rRNA to infer the phylogenetic relationships of six subfamilies proposed by Bouček, and they indicated that Agaonidae that Bouček (1988) referred to was not a monophyletic group [8]; they further revised Agaonidae and pointed out that Agaonidae only contained the subfamily of Agaoninae, and the taxonomic statuses of Epichrysomallinae and Sycophaginae in Chalcidoidea were undetermined. In 2013, Heraty et al. used 233 morphological and two molecular datasets (nuclear ribosomal 18S and 28S D2-D5 expansion regions) to make phylogenetic inference of a variety of wasps in Chalcidoidea and the results showed that the two subfamilies of Epichrysomallinae and Sycophaginae previously undetermined were included in Pteromalidae and Agaonidae, respectively [4]. In 2018, Peters et al. constructed a phylogenetic tree using 3239 homologous genes based on transcriptomic data from 62 species of Chalcidoidea, which showed that Epichrysomallinae was more closely related to Agaonidae than Pteromalidae, but Sycophaginae was not involved in their studies [11]. Therefore, due to different sources of taxonomic evidences (morphology, biological characteristics or molecular evidences), the taxonomy and phylogenetic positions of the two subfamilies of Epichrysomallinae and Sycophaginae, especially the latter, are still unclear, and more data are needed to clarify whether they belong to Agaonidae or Pteromalidae.

The second-generation high-throughput sequencing technology represented by transcriptome RNA sequencing (RNA-seq) can obtain a large-scale sequencing of the transcripts of specific tissues of a certain species. By using bioinformatics tools for splicing assembly, we can quickly obtain almost all the gene coding sequences (CDS) of the specific tissue of the species at a time point. With the advantages of low cost, large data volume, high efficiency and high accuracy, RNA-seq has shown great potential in the field of molecular phylogenetic research and become an effective means for molecular biology research of non-model animals [12,13,14]. The mitochondrial genome (mitogenome) sequences are also widely used in molecular evolution, phylogeny, phylogeography and population genetics because of their advantages, such as small genome size, maternal inheritance, no intron, relatively high evolutionary rate, simple structure, conserved gene content, and rare recombination [15,16,17].

In this study, we obtained conserved single-copy orthologous (SCO) genes in the nucleus based on transcriptomes (*Odontofroggatia galili*, *Walkerella microcarpae*, *Micranisa ralianga*, *Platyneura mayri* and *Encarisa Formosa*) and genomes (another 12 wasp species) from 17 wasp species and mitogenome sequences based on second-generation genome sequencing. Phylogenies of fig wasps were reconstructed based on three different molecular datasets, conserved SCO genes in the nucleus, 13 mitochondrial protein-coding genes (PCGs) and combined genes (SCO genes combined with 13 mitochondrial PCGs) to obtain new evidences to explore the taxonomic status of Epichrysomallinae and Sycophaginae.

## 2. Materials and Methods

### 2.1. Taxon Sampling and Data Collection

A total of 17 wasp species were included in this study (Table 1), including 15 fig wasp species (representing three families and six subfamilies). Four fig wasp species (*O. galili*, *W. microcarpae*, *M. ralianga* and *P. mayri*) were newly sequenced in this study. Data on 11 fig wasp species were from our laboratory and 2 wasp species were download from NCBI. Among the four species, *O. galili*, *W. microcarpae*, and *M. ralianga* were collected from Qinzhou, Guangxi, China (N21°57′, E108°37′), and *P. mayri* was collected from Xishuangbanna, Yunnan, China (N21°41′, E101°25′). The information on the host fig trees was as follows: *O. galili* and *W. microcarpae* were associated to *Ficus microcarpa*, *P. mayri* was associated to *Ficus racemosa*, and *M. ralianga* was associated to *Ficus altissima*. We collected the figs in their natural state of maturity (but fig wasps have not left the fig yet) in the wild. All the fig wasps were collected after they came out from the figs, and then identified by using the SMZ-168 microscope (Motic, China). The identification of fig wasps was based mainly on descriptions and pictures in the literatures [7,18], and also combined with photographs of fig wasps left by Rasplus in Yunnan province. The alive fig wasps were immediately stored in RNA Hold (TransGen, Beijing, China) at −80 °C (for RNA extraction) or in 95% ethanol at −20 °C (for DNA extraction).

### 2.2. RNA Extraction, Transcriptome Sequencing and Assembly

For the RNA extraction of the four fig wasps, we set up two sequencing samples (one female and one male, with 20 to 30 wasps included in each sample) for each wasp species. We picked well-preserved fig wasps in RNA hold and washed them with RNase-free water. We used TransZolUp Plus RNA Kit (TransGen, Beijing, China) to extract RNA for each sample according to the manufacturer’s instructions and finally dissolved the RNA into 40 uL RNase-free water. The concentration and purity of RNA were examined according to the OD values by Thermo Scientific NanoDrop One (ThermoFisher, Waltham, MA, USA). The sequencing libraries were constructed with NEBNext^®^ Ultra™ RNA Library Prep Kit (NEB, Ipswich, MA, USA) and sequenced by second-generation Illumina HiSeq TM2000 platform (Novogen, Tianjin, China).

We obtained at least 6 Gb of raw data for each sample (at least 12 Gb of total data per species). The raw reads were quality controlled by Fastp software [19] and yielded clean reads. For each species, all clean reads were used for *de novo* assembly by using Trinity v2.5.1 [20], with a spliced result file called “Trinity. fasta” generated. Based on the Trinity splicing, Corset program [21] (with default parameters) was employed to cluster the transcripts to obtain the “cluster _all. fasta” file. We selected the longest transcript of each gene in the file of “cluster _all. fasta” as the unique sequence of that gene (also called Unigene).

### 2.3. Prediction of CDS and Identification of SCO Genes

For wasps with non-reference transcriptomes (*O. galili*, *W. microcarpae*, *M. ralianga*, *P*. *mayri,* and *Encarisa Formosa*), TransDecoder v5.5.0 was used to identify the open reading frames (ORFs) of the unigene to obtain the CDS sequences [22]. They were subsequently translated into amino acid sequences (https://web.expasy.org/cgi-bin/translate/dna2aa.cgi), while the CDS and protein sequences of the other species used for analysis were from NCBI or our laboratory.

OrthoMCL v2.0 [23] (with default parameters) was used to construct a local protein database of protein sequences of all species and to perform all-vs-all BLASTP matching to obtain similarity results between protein sequences. The best matched pairs between species (Orthologous pairs) were found via the OrthoMCL Pairs module in OrthoMCL. Finally, all the orthologous homologous proteins were classified and numbered by using Markov Cluster algorithm (MCL) [23]. Eventually, we removed multi-copy homologous proteins and selected families of orthologous proteins with one-to-one relationships (SCO proteins) for subsequent phylogenetic analysis.

### 2.4. DNA Extraction, Library Construction and Sequencing; Mitogenome Assembly and Annotation of 13 PCGs

We picked well-preserved wasps in 95% ethanol and washed them with sterile double distilled water. For each species, we used 30–40 female wasps to extract DNA by using the LiCl/KAc method and dissolved the DNA into 25 uL sterile double distilled water. The concentration and purity of DNA were examined according to the OD values by Thermo Scientific Nano Drop One (ThermoFisher, USA) and DNA integrity was monitored by 1% agarose electrophoresis. Sequencing libraries with an average of insert size of 350 bp were constructed with NEBNext^®^ Ultra™ DNA Library Prep Kit (NEB, USA) and sequenced by second-generation Illumina HiSeq TM2000 platform (Novogen, Tianjin, China). The amount of sequencing data per species was set up at least 4 Gb. Fastp software [19] was used to quality-filtered to obtain the high-quality clean reads.

Mira v4.0.2 [24] and MITObim v1.9.1 [25] were used to assemble the mitogenomes of the four fig wasps, each with a reference mitogenome. The reference species used for *P. mayri* was *Sycophaga agreansis*; the reference species used for *O. galili* was *Sycobia* sp. 2; the reference species used for *W. microcarpae* and *M. ralianga* was *Apocrypta bakeri*. We used Mira v4.0.2 [24] to map clean reads to the reference genome, and MITObim v1.9.1 [25] to assemble the clean reads according to the overlapping regions between the sequences. Then the Mitos web server (http://mitos.bioinf.uni-leipzig.de/index.py) and NCBI ORFfinder (https://www.ncbi.nlm.nih.gov/orffinder/) were used to view and annotate the assembly results. Geneious v2020 [26] was used to assist the poorly assembled fragments of MITObim. For the poorly assembled genomes of *P. mayri* and *M. ralianga*, specific primers were designed to fill the gap regions of the CDS by PCR amplification (Table 2).

### 2.5. Multiple Sequences Alignment, Model Selection and Construction of Phylogeny

For each species, the SCO gene sequences, 13 mitochondrial PCGs sequences, or the combination of these sequences (SCO + mitochondrial PCGs sequences) were respectively concatenated in a specific order into a supergene sequence. The supergene sequences were translated into amino acid sequences using translation software (https://web.expasy.org/cgi-bin/translate/dna2aa.cgi). MAFFT v7.313 [27] was used for multiple sequence alignment. Gblocks v0.91b [28] was used to identify conserved regions and remove unreliably aligned sequences within the datasets. The processed sequences were then used to select the best amino acid substitution model according to the Akaike information criterion with ProtTest v3.4.2 [29]. Setting *Encarisa formosa* as an outgroup, we then performed a phylogeny reconstruction using both Maximum Likelihood (ML) and Bayesian Inference (BI) methods. ML analyses were performed with raxmlGUI v1.5b2 [30], with statistical support for each node estimated using bootstrap, and the rapid bootstrap replicates set to 1000. BI analyses were performed with MrBayes v3.2.6 [31] under the following conditions: 100,000,000 generations, sampled every 1000 generations, a burn-in step for the first 5000 generations. Convergence was deduced for BI phylogenetic tree based on the following metrics: on the one hand, the median standard deviation of split frequencies value was less than 0.01; on the other hand, Tracer v1.7.1 [32] was used to ensure that the effective sample sizes (ESS) value was more than 200. The resulting phylogenetic trees were visualized in FigTree v1.4.4.

## 3. Results

### 3.1. Transcriptomes and Mitogenomes of the Four Newly Sequenced Fig Wasp Species Obtained from High-Throughput Sequencing

In the transcriptome sequencing of *P. mayri*, *O. galili*, *W. microcarpae* and *M. ralianga*, we obtained 103,205,280, 110,176,152, 88,651,962, and 93,048,384 clean reads, respectively. The sequence length distribution of the transcripts and unigenes were showed in Table 3. By using second-generation genome sequencing, for the four species of *P. mayri*, *O. galili*, *W. microcarpae* and *M. ralianga*, we obtained 14,546,375, 25,413,085, 19,783,035, and 19,625,937 clean reads, respectively. Based on these data, we assembled and annotated the mitogenome of the four fig wasps (see Supplementary Tables S1–S4 for detailed annotation).

### 3.2. Identification of SCO Genes and Phylogenetic Analysis

The most important challenges of phylogenomic studies involve different methods for phylogenetic tree reconstruction that can influence the validity of phylogenetic trees [33]. In this study, we identified a total of 145 SCO genes in 17 wasp species. Then we used different molecular datasets including 145 SCO genes, 13 mitochondrial PCGs and combination of these sequences (SCO + mitochondrial PCGs sequences) to reconstruct the phylogenies of fig wasps based on two phylogenetic tree reconstruction methods of ML and BI. In the selection of the model, for the data of the SCO genes and the combined genes, the best-fit model for the amino acid sequences was the JTT + I + G + F model, and the closest substitution model (LG + I + G + F) was selected because the best-fit model was not available in MrBayes v3.2.6. For the 13 mitochondrial PCGs, the MtArt + I + G + F model was the best-fit model for the amino acid sequences, and the closest substitution model (MtREV + I + G + F) was selected because the best-fit model was not available in MrBayes v3.2.6.

**Figure 1:**
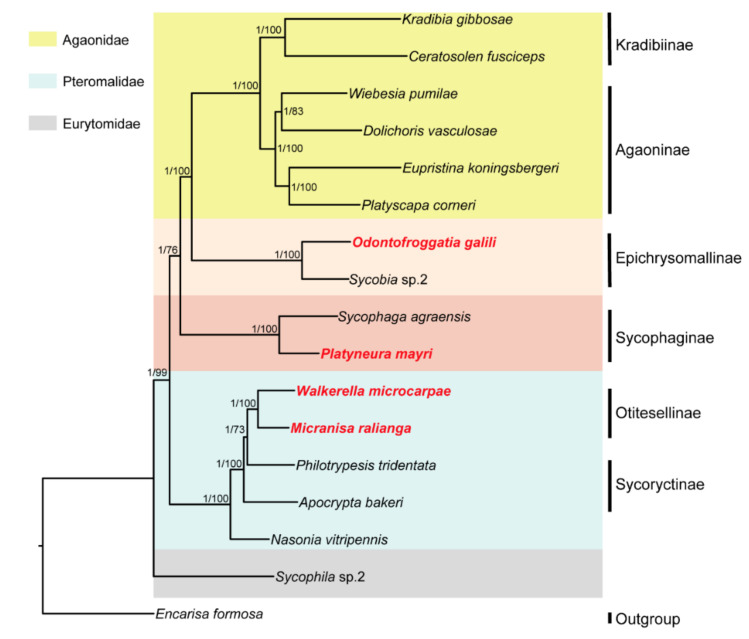
Inferred phylogenetic relationships of 17 chalcidoids based on amino acid (AA) datasets of 145 nuclear SCO genes using ML and BI analyses. *Encarisa Formosa* was used as the outgroup. Bayesian posterior probabilities (BPP) and ML bootstrap values (BP) for each node are shown as: BPP based on AA dataset/BP based on AA dataset, with maxima of 1.00/100.

**Figure 2:**
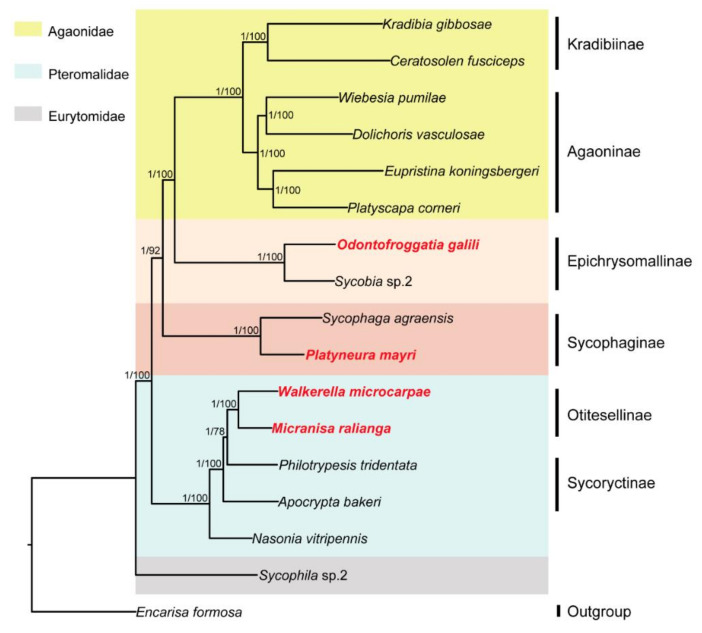
Inferred phylogenetic relationships of 17 chalcidoids based on amino acid (AA) datasets of combined genes (the 145 nuclear SCO genes combined with the 13 mitochondrial PCGs) using ML and BI analyses. *Encarisa Formosa* was used as the outgroup. Bayesian posterior probabilities (BPP) and ML bootstrap values (BP) for each node are shown as: BPP based on AA dataset/BP based on AA dataset, with maxima of 1.00/100.

**Figure 3:**
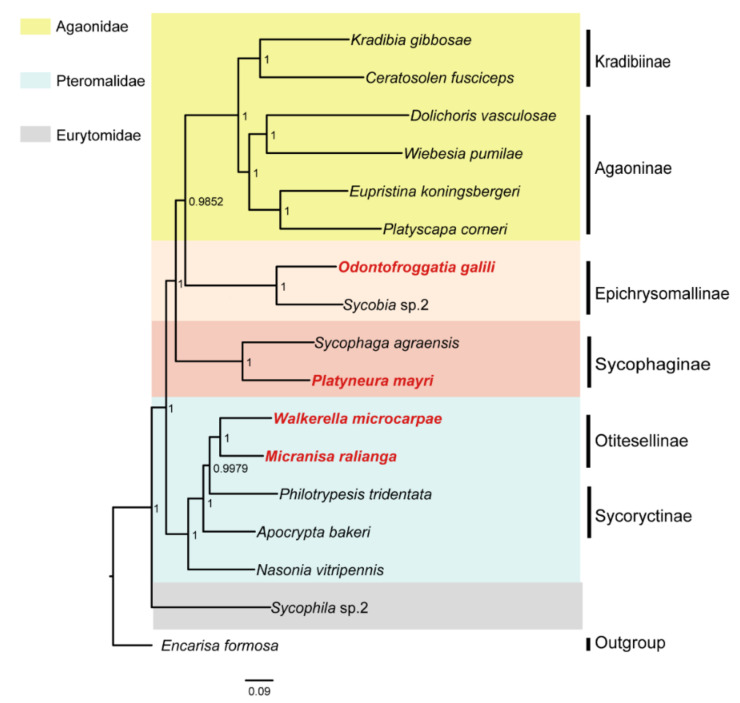
Inferred phylogenetic relationships of 17 chalcidoids based on amino acid (AA) datasets of 13 mitochondrial PCGs using BI analyses. *Encarisa Formosa* was used as the outgroup. Bayesian posterior probabilities (BPP) of each node are shown as BPP based on AA dataset 1.00.

**Figure 4:**
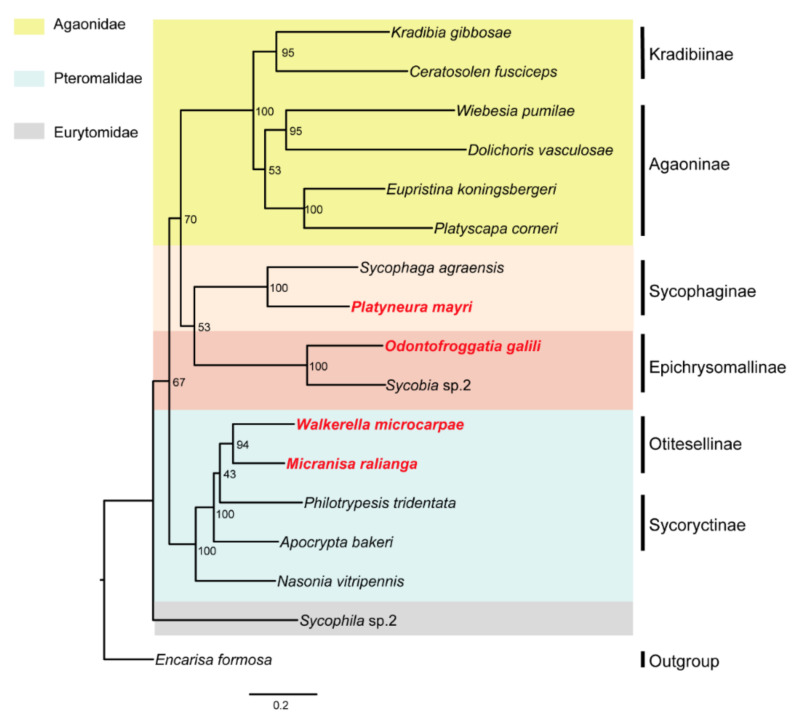
Inferred phylogenetic relationships of 17 chalcidoids based on amino acid (AA) datasets of 13 mitochondrial PCGs using ML analyses. *Encarisa Formosa* was used as the outgroup. Bootstrap values (BP) of each node are shown as BP based on AA dataset 100.

The ML and BI phylogenetic trees constructed with 145 SCO genes were shown in Figure 1; the ML and BI phylogenetic trees constructed with combined genes (the nuclear genes combined with the mitochondrial genes) were shown in Figure 2; the BI tree constructed with 13 mitochondrial PCGs was shown in Figure 3; the ML tree constructed with 13 mitochondrial PCGs was shown in Figure 4. Further analyzing the topology of phylogenetic trees, we are convinced that with the addition of the four newly sequenced species data in this study, the genus affiliations of fig wasps were very clear in all phylogenetic trees we have constructed. *O. galili* and *Sycobia* sp.2 were clustered in one clade of the phylogenetic trees with high node support values (Bayesian posterior probability = 1, Bootstrap value = 100), and these two species belonged to Epichrysomallinae. Similarly, *P. mayri* and *S. agreansis* were clustered in one clade with high nodal support values (Bayesian posterior probability = 1, Bootstrap value = 100), and they belonged to Sycophaginae; *W. microcarpae* and *M. ralianga* belonged to Otitesellinae were clustered in one clade with high nodal support values (Bayesian posterior probability = 1, Bootstrap values = 100 and 94).

When considering the relationships between the subfamilies, our results showed that the ML and BI trees constructed with 145 SCO genes (Figure 1), the ML and BI trees constructed with the combined genes (Figure 2), and the BI tree constructed with 13 mitochondrial PCGs (Figure 3) all displayed the same topology: Epichrysomallinae and Agaonidae formed a clade, which subsequently clustered with Sycophaginae with high statistical support. However, the topology of the ML tree constructed with 13 mitochondrial PCGs (Figure 4) was somewhat divergent, Epichrysomallinae and Sycophaginae clustered together (Bootstrap value = 53), and then this clade was clustered with Agaonidae (Bootstrap value = 70), which was not necessarily reliable given its low statistical support.

## 4. Discussion

Transcriptome sequencing technology can economically and rapidly obtain all RNA information of organisms at a time point and plays an important role in finding molecular datasets for biological research [34]. Mitogenome sequences are also ideal molecular datasets for solving biological phylogeny due to their characteristics of genes without introns, and high evolutionary rate [35]. In this study of fig wasps, focusing on the undetermined taxonomic statuses of Epichrysomallinae and Sycophaginae, we used the SCO genes and mitochondrial PCGs of 17 species based on transcriptome, genome, and mitogenome data to construct phylogenetic trees using ML and BI methods. The final results suggested that both Epichrysomallinae and Sycophaginae were more closely related to Agaonidae, which updated information on the previously unclear phylogenetic statuses of both subfamilies. Compared with previous studies, this is the first time to use transcriptomes, genomes and mitogenome datasets to study the phylogenetic relationship reconstruction of the fig wasps.

Before the emergence of molecular data, the study of taxonomic statuses and phylogenetic relationships primarily relied on morphological characteristics. However, the evolutionary change of morphological characteristics is extremely complicated (even for a short evolutionary time) and phylogenetic trees derived from morphological data are often controversial [36]. Molecular phylogeny is the study of the interrelationships among various groups of organisms in their genealogical and evolutionary processes, by evolutionary study of the structure and function of biological macromolecules (proteins and nucleic acids) [37]. Since the evolutionary changes of DNA and amino acids follow a traceable pattern, it is possible to use a model to compare DNA or protein sequences among different organisms, so molecular phylogeny is expected to clarify problems that has been difficult to be resolved by the classical morphological approaches [38].

However, the incongruences of molecular data have also been frequently observed in phylogenetic trees, possibly due to lack of sufficient phylogenetic information from a single or a few genes [39]. Taking the phylogeny of the two subfamilies (Epichrysomallinae and Sycophaginae) of fig wasps studied in this study as an example, previous results for phylogenetic trees constructed with morphological data indicated that the taxonomic statuses of these two subfamilies had been changing, with Epichrysomallinae firstly being classified to Torymidae, then to Pteromalidae, and finally to Agaonidae by Bouček in 1988 [5,6,7], while Sycophaginae firstly being classified to Torymidae and finally to Agaonidae by Bouček in 1988 [7,8,9,10]. With the use of molecular data, the taxonomic statuses of these two subfamilies became clearer gradually. Initially, the phylogenetic tree constructed with D1 and D2 domains of nuclear 28S rRNA by Rasplus was not able to clarify the taxonomic statuses of these two subfamilies [8]. Subsequently, Heraty et al.’s results based on nucleotide sequences of nuclear ribosomal 18S and 28S D2-D5 expansion regions and 233 morphological data supported that Epichrysomallinae was closely related to Pteromalidae and Sycophaginae was closely related to Agaonidae [4]. Recently, Peters and his colleagues used transcriptomic data to show that Epichrysomallinae are closely related to Agaonidae, but the study did not include data for Sycophaginae [11]. In our study, we construct phylogenetic trees based on 145 SCO genes and 13 mitochondrial PCGs. Compared to the studies of Peters et al. [11], our results not only support their conclusions about Epichrysomallinae that it is closely related to Agaonidae, but our newly added species data about Sycophaginae further confirm that Sycophaginae is closely related to Agaonidae. Our results of Sycophaginae closely related to Agaonidae is consistent with the phylogenetic tree constructed by Heraty [4], even though our conclusions about the status of Epichrysomallinae is inconsistent. Therefore, according to the results of the above analyses, our study supports that both Epichrysomallinae and Sycophaginae are more closely related to Agaonidae. Our results suggest that the phylogenetic relationships of fig wasps cannot be well resolved by mitochondrial data alone, and the combination of nuclear genes, mitogenomes and morphological data will promote the reliability of fig wasp phylogeny. In the future, the addition of more species data will enable us to better understand the phylogenetic relationships of fig wasps.

## 5. Conclusions

In the taxonomic and phylogenetic studies of fig wasps, the taxonomic statuses of the two non-pollinating fig wasp subfamilies of Epichrysomallinae and Sycophaginae are still unclear. We here construct phylogenetic trees with nuclear conserved SCO genes and 13 mitochondrial PCGs genes from 17 wasp species by using the ML and BI methods. Our results show that both Epichrysomallinae and Sycophaginae are closely related to Agaonidae.

## Figures and Tables

**Table 1:** List of the 17 species analyzed in this study.

Species	Subfamily	Family	Accession No. (Mitochondrial Genome)	Accession No. (Genomes or Transcriptomes)
***Platyneura mayri*** *	Sycophaginae *		MW167114	PRJNA672045
***Odontofroggatia galili*** *	Epichrysomallinae *		MW167113	PRJNA671819
***Walkerella microcarpae*** *	Otitesellinae *	Pteromalidae	MW167116	PRJNA672219
***Micranisa ralianga*** *	Otitesellinae *	Pteromalidae	MW167115	PRJNA672141
*Euprisitina koningsbergeri* #	Agaoninae #	Agaonidae	MT947597	PRJNA641212
*Platyscapa corneri* #	Agaoninae #	Agaonidae	MT947604	PRJNA641212
*Dolichoris vasculosae* #	Agaoninae #	Agaonidae	MT947596	PRJNA641212
*Wiebesia pumilae* #	Agaoninae #	Agaonidae	MT947601	PRJNA641212
*Kradibia gibbosae* #	Kradibiinae #	Agaonidae	MT947598	PRJNA641212
*Ceratosolen fusciceps* #	Kradibiinae #	Agaonidae	MT916179	PRJNA494992
*Sycophaga agreansis* *	Sycophaginae *		MT947599	PRJNA641212
*Sycophila* sp.2 *	-	Eurytomidae	MT947603	PRJNA641212
*Sycobia* sp.2 *	Epichrysomallinae *		MT947600	PRJNA641212
*Apocrypta bakeri* *	Sycoryctinae *	Pteromalidae	MT906648	PRJNA641212
*Philotrypesis tridentata* *	Sycoryctinae *	Pteromalidae	MT947602	PRJNA641212
*Nasonia vitripennis*	Pteromalinae	Pteromalidae	EU746609.1, EU746613.1	PRJNA594415
*Encarisa formosa*	Coccophaginae	Aphelinidae	MG813797.1	PRJNA252167

The species in bold represent the fig wasps sequenced in this study. * non-pollinating fig wasps. **#** pollinating fig wasps.

**Table 2:** Specific primers used for PCR amplification of partial mitochondrial regions in this study.

Species	Primers	Sequence (5′–3’)	Annealing Temperature	Targeted CDS Region
*Platyneura mayri*	PF1	ctatataaatttatgaaactatgattaatatctactaatcataaatatattgg	54 °C	The middle part of the *cox1*
	PR1	gataatctaggaggtaataatcaaaatcttatattatttattcgtgg		
*Micranisa ralianga*	MF1	caattaaagttaaacaaattaataagtaaataattgaaattaatattg	50 °C	The middle part of the *nad6*
	MR1	caatttaataataatcattgattttcttatattatatttttaatcatagtag		

**Table 3:** Length distribution of the transcripts and unigenes clustered from the de novo assembly.

	*Platyneura Mayri*		*Odontofroggatia Galili*		*Walkerella Microcarpae*		*Micranisa Ralianga*	
Length Range	Transcript	Unigene	Transcript	Unigene	Transcript	Unigene	Transcript	Unigene
200 bp–500 bp	14,720	4950	38,315	13,104	21,455	6249	24,187	7148
500 bp–1000 bp	11,155	6015	27,822	14,868	13,405	8393	13,659	8435
1000 bp–2000 bp	9368	3799	20,849	8211	11,040	4716	9517	4391
>2000 bp	16,581	5705	19,191	6501	22,073	6541	19,922	6411
Total Number	51,824	20,469	106,177	42,684	67,973	25,899	67,285	26,385
Total Length	99,849,096	35,330,681	130,935,638	49,857,034	133,605,390	42,636,711	127,749,958	42,751,535
Mean Length	1927	1726	1233	1168	1966	1646	1899	1620

## Supplementary Materials

The following are available online at https://www.mdpi.com/2075-1729/11/1/40/s1, Table S1: Summary of the mitogenome of *Platyneura mayri*. Table S2: Summary of the mitogenome of *Odontofroggatia galili*. Table S3: Summary of the mitogenome of *Walkerella microcarpae*. Table S4: Summary of the mitogenome of *Micranisa ralianga*.
