# Supplementary material for: Inferring the Phylogenetic Positions of Two Fig Wasp Subfamilies of Epichrysomallinae and Sycophaginae Using Transcriptomes and Mitochondrial Data
Source: Life (Basel). 2021 Jan 11;11(1):40. doi: 10.3390/life11010040 (PMC7826959; doi:10.3390/life11010040)
Supplement: Supplementary file 1 [file life-11-00040-s001.pdf]

life

MDPI

Supplementary Materials

Inferring the Phylogenetic Positions of Two Fig Wasp Subfamilies of Epichrysomallinae and Sycophaginae Using Transcriptomes and Mitochondrial Data

Table S1. Summary of the mitogenome of *Platyneura mayri*.

Gene	Direction	Location	Size	Anticodon	Start codon	Stop codon	Intergenic nucleotides
rrnL	F	1-1224	1224				32
trnL1	F	1257-1319	63	TAG			-3
nad1	F	1317-2276	960		ATT	TAA	48
trnS2	R	2325-2388	64	TGA			6
cob	R	2395-3516	1122		ATA	TAA	-17
nad6	R	3500-4087	588		ATC	TAA	23
trnP	F	4111-4177	67	TGG			2
trnT	R	4180-4244	65	TGT			58
nad4l	F	4303-4584	282		ATT	TAA	-10
nad4	F	4575-5949	1375		ATT	T	31
trnH	F	5981-6048	68	GTG			-3
nad5	F	6046-7686	1641		ATA	TAA	23
trnF	F	7710-7775	66	GAA			7
trnE	R	7783-7847	65	TTC			-16
cox1	F	7832-9379	1548		ATT	TAA	2
trnL2	F	9382-9447	66	TAA			0
cox2	F	9448-10125	678		ATT	TAA	2
trnD	F	10128-10192	65	GTC			1
trnK	R	10194-10265	72	TTT			37
atp8	F	10303-10473	171		ATT	TAA	0
atp6	F	10474-11151	678		ATG	TAA	2
cox3	F	11154-11945	792		ATG	TAA	9
trnG	F	11955-12020	66	TCC			39
nad3	F	12060-12422	363		ATT	TAA	25
trnC	F	12448-12510	63	GCA			-3
trnN	R	12508-12574	67	GTT			-3
trnR	F	12572-12629	58	TCG			1
trnS1	F	12631-12691	61	TCT			1
trnY	F	12693-12758	66	GTA			0
trnW	R	12759-12824	66	TCA			-2
nad2	R	12823-13653	831		ATA	TAA	-

Table S2. Summary of the mitogenome of *Odontofroggatia galili.*

Gene	Direction	Location	Size	Anticodon	Start codon	Stop codon	Intergenic nucleotides
trnV	F	1-52	52	TAC			9
rrnS	F	62-800	739				14
trnQ	F	815-882	68	TTG			-2
trnA	F	881-940	60	TGC			0
rrnL	F	941-2167	1227				30
trnL1	F	2198-2260	63	TAG			0
nad1	F	2261-3187	927		ATT	TAG	-10
trnS2	R	3178-3239	62	TGA			-1
cob	R	3239-4381	1143		ATG	TAA	0
nad6	R	4382-4919	538		ATG	T	1
trnP	F	4921-4986	66	TGG			-5
trnT	R	4982-5043	62	TGT			1
nad4l	F	5045-5332	288		ATC	TAA	-7
nad4	F	5326-6705	1380		ATG	TAG	158
trnH	F	6864-6926	63	GTG			-3
nad5	F	6924-8615	1692		ATA	TAA	-20
trnF	F	8596-8659	64	GAA			-2
trnE	R	8658-8719	62	TTC			-2
cox1	F	8718-10250	1533		ATA	TAG	-5
trnL2	F	10246-10309	64	TAA			0
cox2	F	10310-10987	678		ATC	TAA	2
trnK	R	10990-11051	62	TTT			0
trnD	F	11052-11114	63	GTC			28
atp8	F	11143-11304	162		ATT	TAA	-7
atp6	F	11298-11969	672		ATG	TAA	2
cox3	F	11972-12760	789		ATG	TAA	72
nad3	F	12833-13189	357		ATA	TAA	-8
trnR	F	13182-13242	61	TCG			-4
trnN	R	13239-13300	62	GTT			-2
trnS1	F	13299-13354	56	TCT			0
trnC	F	13355-13418	64	GCA			0
trnY	F	13419-13483	65	GTA			-3
nad2	R	13481-14359	879		ATT	TAG	141
trnG	F	14501-14563	63	TCC			0
trnI	F	14564-14628	65	GAT			2
trnW	F	14631-14694	64	TCA			-

Table S3. Summary of the mitogenome of *Walkerella microcarpae.*

Gene	Direction	Location	Size	Anticodon	Start codon	Stop codon	Intergenic nucleotides
trnR	R	1-68	68	TCG			1
trnS2	R	70-136	67	TGA			-1
trnI	F	136-204	69	GAT			8
trnM	F	213-280	68	CAT			0
trnV	F	281-346	66	TAC			1
rrnS	F	348-1123	776				-6
trnA	F	1118-1180	63	TGC			-5
rrnL	F	1176-2533	1358				-43
trnL1	F	2491-2553	63	TAG			-3
nad1	F	2551-3468	918		ATA	TAA	-33
cob	R	3436-4542	1107		ATT	TAA	-53
nad6	R	4490-5053	564		ATA	TAA	35
trnP	F	5089-5156	68	TGG			27
trnT	R	5184-5236	53	TGT			6
nad4l	F	5243-5530	288		ATT	TAA	-25
nad4	F	5506-6879	1374		ATT	TAG	-10
trnH	F	6870-6932	63	GTG			-3
nad5	F	6930-8291	1362		ATT	TAA	310
trnF	F	8602-8667	66	GAA			-2
trnE	R	8666-8730	65	TTC			6
cox1	F	8737-10269	1533		ATG	TAA	8
trnL2	F	10278-10345	68	TAA			1
cox2	F	10347-11030	684		ATG	TAA	8
trnK	F	11039-11108	70	TTT			6
trnD	F	11115-11181	67	GTC			0
atp8	F	11182-11346	165		ATT	TAA	-7
atp6	F	11340-12014	675		ATG	TAA	-1
cox3	F	12014-12797	784		ATG	T	1
trnG	F	12799-12865	67	TCC			54
nad3	F	12920-13264	345		ATC	TAA	10
trnC	F	13275-13339	65	GCA			7
trnS1	F	13347-13406	60	TCT			63
trnS1	F	13470-13529	60	TCT			6
trnY	F	13536-13603	68	GTA			4
trnW	R	13608-13674	67	TCA			1
nad2	R	13676-14710	1035		ATA	TAA	58
trnQ	R	14769-14835	67	TTG			-

Table S4. Summary of the mitogenome of *Micranisa ralianga.*

Gene	Direction	Location	Size	Anticodon	Start codon	Stop codon	Intergenic nucleotides
trnM	F	1-68	68	CAT			1
trnV	F	70-133	64	TAC			26
rrnS	F	160-904	745				0
trnA	F	905-967	63	TGC			16
rrnL	F	984-2415	1432				-24
trnL1	F	2392-2458	67	TAG			0
nad1	F	2459-3374	916		ATA	T	0
trnQ	F	3375-3442	68	TTG			1
trnQ	F	3444-3511	68	TTG			0
trnR	R	3512-3576	65	TCG			0
trnS2	R	3577-3642	66	TGA			9
cob	R	3652-4779	1128		ATG	TAA	-1
nad6	R	4779-5354	576		ATT	TAA	-8
trnP	F	5347-5415	69	TGG			0
trnT	R	5416-5480	65	TGT			2
nad4l	F	5483-5756	274		ATT	T	1
nad4	F	5758-7114	1357		ATT	T	0
trnH	F	7115-7179	65	GTG			-20
nad5	F	7160-8857	1698		ATA	TAA	-1
trnF	F	8857-8921	65	GAA			0
trnE	R	8922-8985	64	TTC			-6
cox1	F	8980-10518	1539		ATA	TAA	24
trnL2	F	10543-10608	66	TAA			0
cox2	F	10609-11286	678		ATT	TAA	31
trnK	R	11318-11388	71	TTT			2
trnD	F	11391-11462	72	GTC			0
atp8	F	11463-11627	165		ATC	TAA	36
atp6	F	11664-12336	673		ATG	T	-3
cox3	F	12334-13120	787		ATT	T	0
trnG	F	13121-13185	65	TCC			54
nad3	F	13240-13554	315		ATT	TAA	-2
trnC	F	13553-13618	66	GCA			-3
trnN	R	13616-13681	66	GTT			88
trnS1	F	13770-13833	64	TCT			9
trnY	F	13843-13912	70	GTA			11
nad2	R	13924-14931	1008		ATA	TAA	2
trnI	F	14934-15005	72	GAT			-
